# Prediction of Early Response to Immune Checkpoint Inhibition Using FDG-PET/CT in Melanoma Patients

**DOI:** 10.3390/cancers13153830

**Published:** 2021-07-29

**Authors:** Ken Kudura, Florentia Dimitriou, Lucas Basler, Robert Förster, Daniela Mihic-Probst, Tim Kutzker, Reinhard Dummer, Joanna Mangana, Irene A. Burger, Michael C. Kreissl

**Affiliations:** 1 Department of Nuclear Medicine, University Hospital Zurich, 8091 Zurich, Switzerland; irene.burger@usz.ch; 2 Department of Dermatology, University Hospital Zurich, 8091 Zurich, Switzerland; florentia.dimitriou@usz.ch (F.D.); reinhard.dummer@usz.ch (R.D.); johanna.mangana@usz.ch (J.M.); 3 Center for Proton Therapy, Paul Scherrer Institute, 5232 Villigen, Switzerland; lucas.basler@psi.ch; 4 Institute of Radiation Oncology, Cantonal Hospital Winterthur, 8400 Winterthur, Switzerland; robert.foerster@ksw.ch; 5 Institute of Pathology and Molecular Pathology, University Hospital Zurich, 8091 Zurich, Switzerland; daniela.mihic@usz.ch; 6 Faculty of Applied Statistics, Humboldt University Berlin, 10117 Berlin, Germany; tim.kutzker@hu-berlin.de; 7 Department of Nuclear Medicine, Cantonal Hospital Baden, 5404 Baden, Switzerland; 8 Department of Radiology and Nuclear Medicine, Division of Nuclear Medicine, University Hospital Magdeburg, 39120 Magdeburg, Germany; michael.kreissl@med.ovgu.de

**Keywords:** positron emission tomography computed tomography, melanoma, immunotherapy, biomarkers, outcome prediction

## Abstract

**Simple Summary:**

Melanoma has become the most rapidly increasing cancer in Caucasian populations, causing 90% of skin cancer mortality. FDG-PET/CT has been recommended by the European 2019 guidelines for melanoma for staging and treatment response assessment in advanced melanoma highlighting the need for new outcome predictive biomarkers. In the context of melanoma, the evidence on the predictive value of semiquantitative parameters derived from FDG-PET/CT is still very limited. We here provide evidence, in a large cohort of metastatic melanoma patients, that FDG-PET/CT can be used to predict the early response to immune checkpoint inhibition. On a patient-basis, total tumor volume and semiquantitative parameters, such as total metabolic tumor volume MTV and total lesion glycolysis TLG of all metastases three months after treatment start are promising predictive biomarkers for the outcome in metastatic melanoma patients. Also, early complete response on a metastasis- and patient-level seems to be predictive for lasting complete response.

**Abstract:**

We aimed to investigate, whether ^18^F*-*2-fluoro-2-desoxy-D-glucose positron emission tomography/computed tomography (FDG-PET/CT) scans performed at baseline (time point 0; TP 0) and three months after initiation of immunotherapy (time point 1; TP 1) can be used on a metastasis- and patient-level to predict the response to immune-checkpoint inhibition using FDG-PET/CT six months after treatment start (time point 2; TP 2) in metastatic melanoma patients. This single-center retrospective study considered metastatic melanoma patients treated with immune checkpoint inhibition from TP 0 to TP 2. An analysis on a metastasis- and patient-level was carried out. Tumor volume, standardized uptake values SUV (mean, maximum, and peak), metabolic tumor volume MTV and total lesion glycolysis TLG of each included metastasis were recorded at each time point, respectively TP 0, TP 1 and TP 2. Total tumor volume, total metabolic tumor volume and total lesion glycolysis per patient were also calculated at TP 0, TP 1 and TP 2. Treatment response was assessed at metastasis- and patient-level based on FDG-PET/CT scans at TP 2. 612 melanoma metastases in 111 patients were included. The analysis on a metastasis-level showed that metastatic SUVpeak at TP 1 and volume variation between TP 0 and TP 1 were the strongest negative predictive biomarkers for response. However, at TP 0, metastatic SUVmean and SUVpeak indicated a low negative prediction power, whereas initial metastatic volume was not a predictive biomarker. Also, melanoma metastases located in bone structures had a negative influence on the outcome at TP 2, particularly in women. The analysis on a patient-level showed, that total tumor volume, total metastatic tumor volume and total lesion glycolysis of all metastases three months after treatment initiation were strong negative predictive biomarkers for response to immunotherapy six months after initiation. Age and female sex were also found to be negative predictive biomarkers with lower predictive power. Interestingly, total tumor volume at TP 0 and number of metastases at TP 0 as well as the occurrence of early immune-related adverse events between TP 0 and TP 2 did not have any predictive value for early treatment response. FDG-PET/CT performed for treatment response assessment three months after initiation of immune checkpoint inhibition in metastatic melanoma patients can also be used to predict early response to treatment. On a metastasis-level SUV peak and volume variation of metastases are strong outcome predictive biomarkers. On a patient-level total tumor volume and semiquantitative parameters such as total metabolic tumor volume MTV and total lesion glycolysis TLG of all metastases are promising outcome predictive biomarkers. Also, early complete response on a metastasis- and patient-level seems to be predictive for lasting complete response.

## 1. Introduction

Cutaneous melanoma has become the most rapidly increasing cancer in Caucasian populations [1], causing 90% of skin cancer mortality [2]. The recent introduction of immune checkpoint inhibitors (ICI) has revolutionized the treatment of advanced melanoma [3], leading to a significantly higher life expectancy in treated melanoma patients [4]. However, an accurate patient selection for immunotherapy remains challenging given not only the heterogeneous mutation profile of cutaneous melanoma [5], but also the risk of exposing treatment-resistant patients to higher immune-related toxicity [6,7]. A personalized approach with individual biomarkers predictive of treatment response thus appears necessary. In order to predict the response to immunotherapy several outcome predictive biomarkers based on the histopathology of the primary tumor (i.e., tumor thickness, ulceration, mitotic rate) [8], standard blood samples (i.e., lactate dehydrogenase LDH, calcium-binding protein B S100B) [9] or clinical aspects (i.e., sentinel lymph node involvement, anatomical site of primary tumor, gender or age) [10] have been already discussed in the literature. However the 2019 European guidelines for cutaneous melanoma recommending FDG-PET/CT for staging and treatment response assessment in advanced melanoma have highlighted the need for further individual outcome predictive biomarkers based on hybrid imaging [2].

The value of semiquantitative parameters, such as standardized uptake values SUV on FDG-PET/CT scans to predict treatment response has been already extensively discussed in the literature for several malignancies. High primary SUVmax has been associated with poor outcome and higher risk of recurrence in patients with breast cancer [11]. In oral cancer patients high primary SUVmax has also shown a significantly higher risk of local recurrence and occult nodal metastases [12].

However, in the context of melanoma, the evidence on the predictive value of semiquantitative parameters is still very limited since only a few investigations with small cohorts have been performed so far, highlighting the urgent need for further investigations with larger populations [13,14,15].

We aimed to investigate, whether metastatic volume and semiquantitative parameters on FDG-PET/CT scans performed at baseline TP 0 and three months after initiation of immunotherapy TP 1 can be used on a metastasis- and patient-level to predict the outcome six months after initiation of immune checkpoint inhibition TP 2 in a large cohort of metastatic melanoma patients.

## 2. Methods

### 2.1. Patient Cohort

In this single-center retrospective study patients with histopathologically proven metastatic melanoma treated with either single checkpoint-inhibition (anti-PD-1) or dual checkpoint-inhibition (anti-PD-1/anti-CTLA-4) between 2013 and 2019 at the Department of Dermatology of the University Hospital Zurich in Switzerland were considered. FDG-PET/CT scans performed in clinical routine at regular intervals were mandatory for patient inclusion. In order to minimize the risk of misinterpretation through pseudoprogression FDG-PET/CT scans before starting immunotherapy (i.e., baseline timepoint TP 0), 3 and 6 months after treatment initiation performed for response assessment under immune checkpoint inhibition (i.e., first follow up time point TP 1 and second follow up time point TP 2) were mandatory. The average interval between FDG-PET/CT scan at TP 0 and TP 1 was 107.1 days and 93.1 days between TP 1 and TP 2. All included patients consented the use of their clinical data for research purposes. This study was approved by the local ethics committee (protocol code KEK-ZH-Nr: 2014-0193) and conducted in compliance with Good Clinical Practice GCP-rules and the Declaration of Helsinki.

### 2.2. Clinical Data

Clinical data (such as gender, age, prior treatment, histopathology of primary tumor, anatomical site of metastasis, immunotherapy agent, date of treatment start, occurrence of immune-related adverse events under immune checkpoint inhibition and clinical follow up of treatment, i.e., local treatment of metastasis, treatment abort or patient death) were provided in kind cooperation with the Department of Dermatology based on internal clinical records.

### 2.3. FDG-PET/CT Acquisition

The considered FDG-PET/CT scans were all performed in clinical routine at the Department of Nuclear Medicine of the University Hospital Zurich, according to the department’s standard protocol. Given the wide time window (2013–2019) four different PET/CT scanners by General Electric (GE, Boston, Massachusetts, United States of America) were used over time, i.e., Discovery ST16 und VCT (2013–2017), Discovery 690 (2013–2019) and Discovery DMI (2017–2019). All scanners were calibrated as well as cross calibrated against each other. Furthermore, the four single-center PET/CT scanners were numbered from one to four in the listed order above and included in the statistical analysis in order to investigate whether the use of four different single-center PET/CT scanners had an influence on the measurement of semiquantitative parameters.

All examinations were performed from the vertex of the skull to the thighs in supine position. Whole body FDG-PET/CT scans only if the primary melanoma was located in the lower extremities. A CT scan without contrast medium was performed first for attenuation correction (tube potential 120 kV; tube current modulation between 15 and 80 mA; matrix size 512 × 512; field of view 50 cm; slice thickness 3.75 mm) immediately followed by the PET acquisition (matrix size 256 × 256; field of view 70 cm) in time-of-flight TOF technique.

Patients were asked to fast at least 4 h prior the intravenous ^18^F-FDG-administration. A blood glucose level below 160 mg/dL at the time of ^18^F-FDG injection was mandatory. Image acquisition began 60 min after the administration of a body mass index (BMI)-adapted ^18^F-FDG dose.

### 2.4. Lesion Segmentation

All included melanoma metastases were retrospectively delineated on coregistered CT- and PET-images at all three time points by two independent physicians using a manual 3D-contouring tool.

The standardized uptake values (SUV mean, maximum and peak), as well as metabolic tumor volume MTV and total lesion glycolysis TLG of each included metastasis were extracted from the same volume of interest VOI surrounding the whole metastasis on PET images reconstructed with ordered subset expectation maximization OSEM (3 iterations, 16 subsets) and a threshold set at 42% of the SUVmax.

The metastatic volume was measured using a VOI surrounding the whole metastasis on native computed tomography scans. The CT-based contours could be manually corrected by matching the lesion borders on CT- and PET-images.

As part of the analysis on a patient-level, total tumor volume, total metabolic tumor volume MTV and total lesion glycolysis TLG of all metastases were determined.

Newly occurred metastases at TP 1 were not segmented, but only documented (i.e., onset of new metastasis).

In order to optimize the accuracy and also the reproducibility of the measurements using a manual 3D-contouring tool following exclusion criteria were applied in accordance with the aim of the investigation.

Cardiac metastases were not included due to the surrounding physiological activity of the left ventricle and difficult morphological assignment on native computed tomography scans (*n* = 3).

Since the aim of the investigation was to predict the response to systemic immunotherapy and not local treatment locally treated or resected metastases during treatment were excluded (*n* = 71).

Finally, very small metastases at baseline (metastatic volume < 0.5 mL) were also not included (*n* = 18) in order to ensure accurate measurements using a manual 3D-contouring tool by two independent readers.

All segmented metastases could be divided according to their anatomical site into four groups: bone, liver, lung and soft tissue (including cutaneous/subcutaneous, muscular and lymph nodes metastases).

### 2.5. Treatment Response Assessment

#### 2.5.1. Analysis at Metastasis-Level

The response to immunotherapy was assessed at metastasis-level six months after treatment initiation TP 2 in order to minimize the risk of misinterpretation through pseudoprogression at 3 months TP 1.

A metastatic complete response CR was reached when the metastasis disappeared (i.e., not measurable).

A metastasis with a decrease ≥ 30% in the sum of diameters showed partial response PR.

A metastasis with an increase ≥ 20% in the sum of diameters showed progressive disease PD.

A metastasis was reported as stable disease SD in any case not qualifying for partial response or progressive disease.

Subsequently, all metastases were dichotomized into two groups, respectively progressive metastases vs. non progressive metastases with clinical benefit CB 6 months after immunotherapy initiation (i.e., complete response CR; partial response PR; stable disease SD).

Finally, we investigated whether volume and standardized uptake values of metastases at TP 0 and TP 1 can be used to predict whether metastases benefit from treatment or show progressive disease six months after immunotherapy at TP 2.

#### 2.5.2. Analysis at Patient-Level

The response to immunotherapy was also assessed at patient-level six months after treatment initiation TP 2 in order to minimize the risk of misinterpretation through pseudoprogression at 3 months TP 1.

A complete response CR was reached when the metastases disappeared (i.e., not measurable).

A partial response PR was reached when the metastases showed a decrease ≥ 30% in the sum of diameters.

A progressive disease PD was reached when metastases showed an increase ≥ 20% in the sum of diameters.

A stable disease SD was reached in any case not qualifying for partial response or progressive disease.

Subsequently, all included patients were dichotomized into two groups, respectively patients with clinical benefit CB 6 months after immunotherapy initiation (i.e., complete response CR; partial response PR; stable disease SD) vs. patients with no clinical benefit (progressive disease PD).

Finally, we investigated whether total tumor volume, total metabolic tumor volume and total lesion glycolysis of all metastases at TP 0 and TP 1 can be used to predict whether patient would benefit from treatment or show progressive disease six months after immunotherapy at TP 2.

#### 2.5.3. Statistical Analysis

Statistical analysis was performed in R (version 3.3.3, R core team). An analysis of variance (ANOVA) was carried out in order to investigate, whether the use of four different single-center PET/CT scanners had an influence on the semiquantitative parameters. For outcome prediction at metastasis-level a binominal logistic regression was used with patient and anatomical site of metastasis as random effects independent of one another. For outcome prediction on a patient-level, a stepwise regression (backward selection) was used. Statistical significance was accepted at *p* < 0.050.

## 3. Results

### 3.1. Baseline Characteristics of Patient Cohort

One hundred and eleven (111) metastatic melanoma patients were included (69.4 % male, *n* = 77; 30.6% female, *n* = 34), mostly treated with single checkpoint inhibition (84.7%, *n* = 94) from TP 0 to TP 2. 61% of all patients (*n* = 68) had already been pretreated at TP 0 (with single or dual checkpoint inhibition, BRAF-inhibitor, MEK-inhibitor or chemotherapy), while 39% of all patients (*n* = 43) were untreated at TP 0. Among the 111 included metastatic patients 17 patients had melanoma metastases of unknown primary, 85 patients metastases of cutaneous melanoma and nine patients metastases of non-cutaneous melanoma (Table 1).

**Figure 1:**
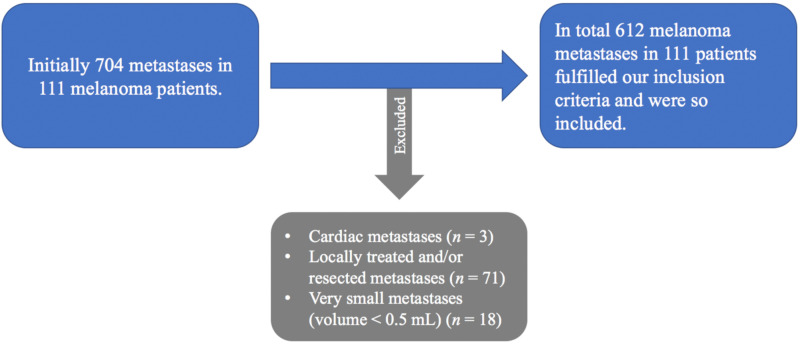
Exclusion criteria. In order to optimize the accuracy and also the reproducibility of the measurements using a manual 3D-contouring tool in accordance with the aim of the investigation 92 melanoma metastases were excluded. In total, 612 melanoma metastases fulfilled our criteria.

Seven hundred and four (704) metastases were documented among the 111 included patients. In total, 612 melanoma metastases fulfilled our inclusion criteria and were divided into four groups according to anatomical site at TP 0: 319 soft tissue, 137 lung, 111 liver/spleen and 45 bone metastases (Figure 1).

### 3.2. Analysis

Four different single-center PET/CT scanners were used for the measurement of semiquantitative parameters. In addition to calibration and cross calibration of the four single-center PET/CT scanners an analysis of variance (ANOVA) was carried out to exclude any effect on semiquantitative parameters by the different scanners. Therefore, the SUVmaximum at TP 0, TLG at TP 0, as much as the PET/CT scanner used at TP 0 were considered. Since no significant scanner effect was observed on SUVmax (*p* = 0.950) or TLG (*p* = 0.179), the semiquantative parameters of the four different single-center PET/CT scanners can be pooled and the variable scanner removed for the following analysis at metastasis- and patient-level.

### 3.3. Analysis at Metastasis-Level

#### 3.3.1. Metastatic Volume at Baseline TP 0

**Figure 2:**
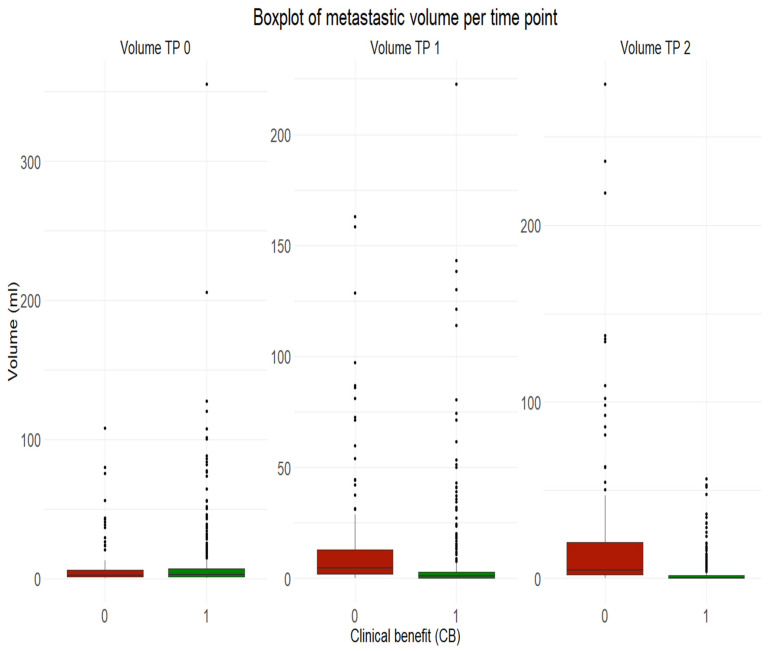
Boxplot of metastatic volume per time point. In green (1) non progressive melanoma metastasis with clinical benefit six months after treatment. In red (0) progressive melanoma metastasis six months after treatment. First column from left: Metastatic volume at baseline TP 0. Column in the middle: Metastatic volume three months after initiation of immunotherapy TP 1. First column from right: Metastatic volume six months after initiation of immunotherapy TP 2.

The median volume of progressive metastases was 2.40 mL (inter-quartile range IQR 1.28–5.93). The median volume of non-progressive metastases with clinical benefit was 2.66 mL (IQR 1.47–6.88) (Figure 2).

#### 3.3.2. Standardized Uptake Values at Baseline TP 0

**Figure 3:**
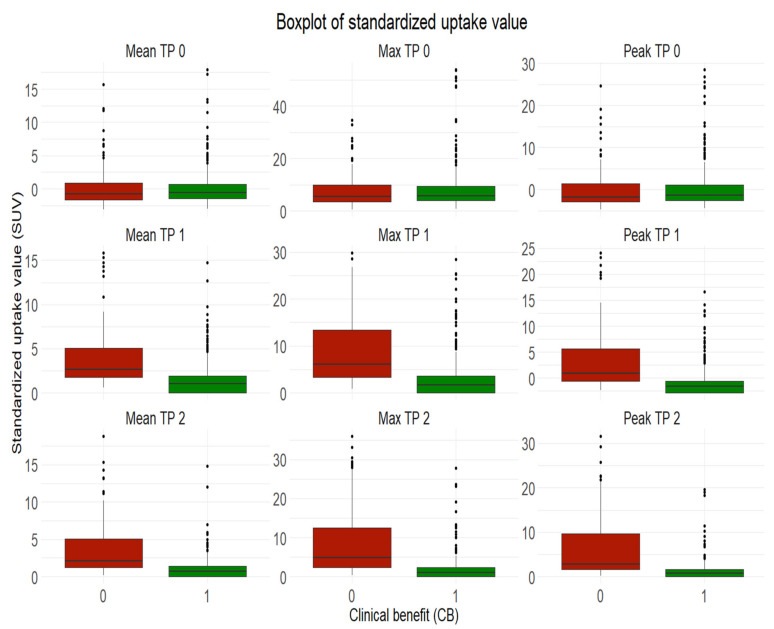
Boxplot of standardized uptake value per time point SUVmean (first column from left), SUVmax (column in the middle) and SUVpeak (first column from right). In green non progressive melanoma metastasis with clinical benefit six months after treatment. In red progressive melanoma metastasis six months after treatment. First raw from the top: at baseline TP 0. Second raw: three months after initiation of immunotherapy TP 1. First raw from the bottom: six months after initiation of immunotherapy TP 2.

Following median values were observed in progressive metastases: SUV mean 0.8 (IQR 0.7–1.6); SUVmax 5.4 (IQR 3.6–9.9); SUVpeak 1.8 (IQR 1.44–2.8). Following median values were observed in non-progressive metastases with clinical benefit: SUV mean 0.7 (IQR 0.6–1.43); SUVmax 5.7 (IQR 4.0–9.4); SUVpeak 1.3 (IQR 1.1–2.6) (Figure 3).

#### 3.3.3. Metastatic Volume 3 Months after Treatment Initiation TP 1

The median volume of progressive metastases was 4.77 mL (IQR 1.85–12.98). The median volume of non-progressive metastases with clinical benefit was 0.89 mL (IQR 0.00–2.99) (Figure 2).

#### 3.3.4. Standardized Uptake Values 3 Months after Treatment Initiation TP 1

Following median values were observed in progressive metastases: SUV mean 2.6 (IQR 1.8–5.1), SUVmax 6.0 (IQR 3.3–13.4); SUVpeak 0.9 (IQR 0.6–5.7). Following median values were observed in non-progressive metastases with clinical benefit: SUV mean 1.0 (0.0–1.9); SUVmax 1.7 (0.0–3.6); SUVpeak 1.6 (0.6–2.8) (Figure 3).

#### 3.3.5. Metastatic Volume 6 Months after Treatment Initiation TP 2

The median volume of progressive metastases was 4.89 mL (2.03–20.38). The median volume of non-progressive metastases with clinical benefit was 0.32 mL (0.00–1.56) (Figure 2).

#### 3.3.6. Standardized Uptake Values 6 Months after Treatment Initiation TP 2

Following median values were observed in progressive metastases: SUV mean 2.1 (IQR 1.3–5.0); SUVmax 4.9 (IQR 2.3–12.5); SUVpeak 2.8 (IQR 1.7–9.7). Following median values were observed in non-progressive metastases with clinical benefit: SUV mean 0.7 (IQR 0.0–1.4); SUVmax 1.0 (IQR 0.0–2.4); SUVpeak 0.8 (IQR 0.0–1.6) (Figure 3).

#### 3.3.7. Treatment Response

Six hundred and twelve (612) melanoma metastases were included at baseline. Three months after initiation of immune checkpoint inhibition 129 metastases showed complete response, 238 metastases partial response, 125 metastases stable disease and 120 metastases progressive disease. In order to minimize the risk of misinterpretation through pseudoprogression at that time a subsequent treatment response assessment was performed three months later (i.e., six months after treatment start) with following results: 170 metastases in complete response, 195 metastases in partial response, 175 metastases in stable disease and 72 metastases in progressive disease.

Metastases showing stable disease at TP 2 displayed the group with the greatest increase in number of incoming metastases from TP 1 to TP 2 (+40%), mostly metastases showing partial response at TP 1. Metastases displaying complete response at TP 2 constituted the group with the second greatest increase in number of incoming metastases from TP 1 to TP 2 (+32%), mostly metastases showing partial response at TP 1.

**Figure 4:**
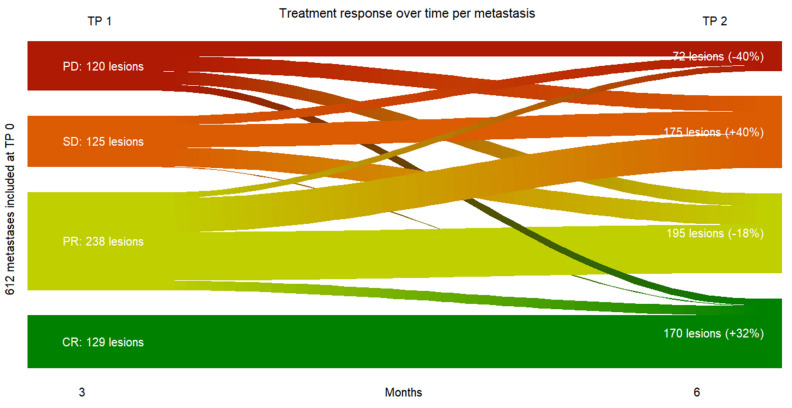
Sankey plot of treatment response over time from TP 0 to TP 2 per metastasis. In total, 612 melanoma metastases were included at TP 0. From the top to the bottom: Treatment response per metastasis. Progressive Disease PD in red; Stable Disease SD in orange; Partial Response PR in lime-green; Complete Response CR in green. From left to right: Time point baseline TP 0; three months after initiating immunotherapy TP 1; six months after initiation immunotherapy TP 2.

In total, 19.6 % (*n* = 120) of all included metastases were progressive metastases at TP 1 vs. only 11.8% % (*n* = 72) at TP 2, which in turn meant 80,4% (*n* = 500) at TP 1 and 88.2% (*n* = 540) at TP 2 of all metastases were non progressive metastases with clinical benefit (Figure 4).

### 3.4. Prediction of Outcome at TP 2

#### 3.4.1. At TP 0

SUV peak (*p*= 0.013) and SUVmean (*p*= 0.016) of melanoma metastases showed a lower prediction power, whereas SUVmax (*p*= 0.366) and initial volume (*p*= 0.217) of metastases were not predictive biomarkers.

#### 3.4.2. At TP 1

In order to assess metastatic volume variation in the first three months of treatment, we introduced a new feature named volume ratio TP 1 to TP 0, defined as metastatic volume at TP1 divided by metastatic volume at TP 0.

**Figure 5:**
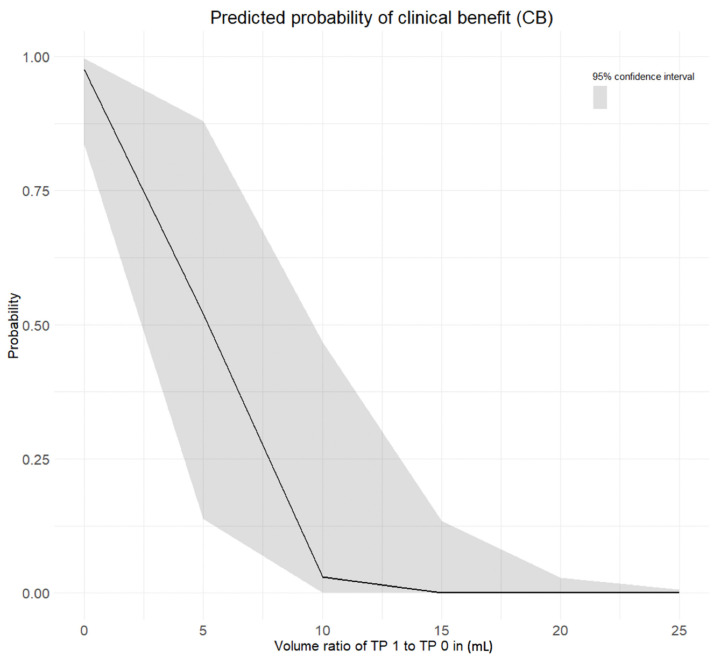
Predicted probability of clinical benefit at TP 2 based on volume ratio TP1 to TP 0 (in ml). On the x-axis: Volume ratio TP 1 to TP 0 (in ml). On the y-axis: Probability of clinical benefit at TP 2.

The volume ratio TP 1 to TP 0 showed a strong negative prediction power for treatment outcome at TP 2 (*p* = 1.3 × 10^−4^). The smaller the volume ratio TP 1 to TP 0 the higher the probability of clinical benefit at TP 2 (Figure 5).

Among the metabolic features SUVpeak owned the strongest negative prediction power (*p* = 1.3 × 10^−4^), while SUVmax (*p* = 0.844) and SUVmean (*p* = 0.758) were not predictive biomarkers. The onset of new metastases at TP 1 displayed a lower negative prediction power (*p* = 0.032).

### 3.5. Clinical Parameters

Although bone metastases represented by far the smallest entity of all included metastases at TP 0, their presence seemed to have a negative influence on the outcome at TP 2, especially in women showing the lowest likelihood of clinical benefit compared to other metastasis entities. A bone metastasis in women had a 6% lower likelihood to display a clinical benefit compared to men.

### 3.6. Analysis on a Patient-Level

#### 3.6.1. Treatment Response

111 metastatic melanoma patients were included at baseline. Three months after initiation of immune checkpoint inhibition, three patients showed complete response, 27 patients partial response, 55 patients stable disease and 26 patients progressive disease. In order to minimize the risk of misinterpretation through pseudoprogression at that time a subsequent treatment response assessment was performed three months later (i.e., six months after treatment start) at TP 2 with following results: nine patients in complete response, 10 patients in partial response, 82 patients in stable disease and 10 patients in progressive disease.

Patients displaying complete response at TP 2 constituted the group with the greatest increase from TP 1 to TP 2 (+200%). The group of patients showing stable disease at TP 2 was the largest group and showed an increase from TP 1 to TP 2 of 49%. The group of patients with progressive disease at TP 2 was the group that showed the largest decrease from TP 1 to TP 2 (−62%).

**Figure 6:**
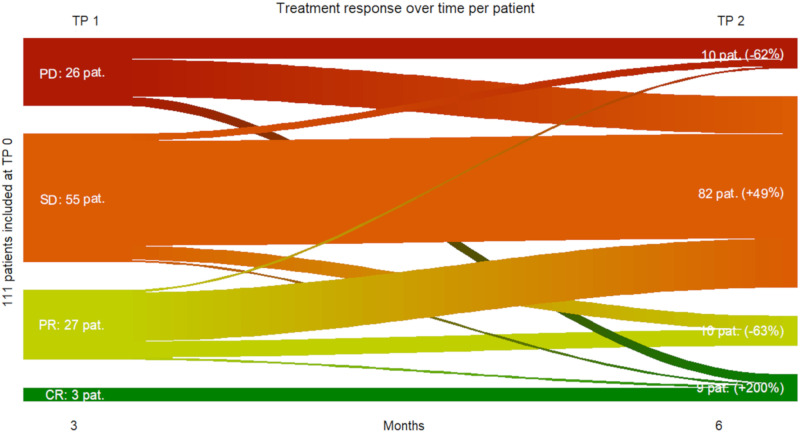
Sankey plot of treatment response over time from TP 0 to TP 2 per patient. In total 111 metastatic melanoma patients were included at TP 0. From the top to the bottom: Treatment response per patient. Progressive Disease PD in red; Stable Disease SD in orange; Partial Response PR in lime-green; Complete Response CR in green. From left to right: Time point baseline TP 0; three months after initiating immunotherapy TP 1; six months after initiation immunotherapy TP 2.

In total, according to our definition, 90% of all included metastatic melanoma patients showed a clinical benefit from immune checkpoint inhibition (e.g., no disease progression under treatment) six months after treatment initiation, while 10% had progressive disease (Figure 6).

#### 3.6.2. Outcome Prediction

In order to avoid multicolinearity effects, a backward regression was performed. The following parameters were initially considered for outcome prediction at TP 2 using a backward regression: age, gender, initial number of metastases at TP 0, occurrence of immune-related adverse events under immune checkpoint inhibition between TP 0 and TP 2 (*n* = 38 patients, 34.2%), total tumor volume TV at TP 0 and TP1, total metabolic tumor volume MTV at TP 0 and TP 1, total lesion glycolysis of all metastases at TP 0 and TP 1. Total TV at TP 1 (*p* = 0.007), total MTV at TP 1 (*p* = 0.006) and TLG of all metastases at TP 1 (*p* = 0.005) were strong predictive biomarkers for response to immune checkpoint inhibition at TP 2. Age (*p* = 0.038) and female sex (*p* = 0.047) were also negative predictive biomarkers with lower prediction power. Interestingly, the initial number of metastases, occurrence of immune-related adverse events and initial tumor volume at TP 0 did not show any prediction power for the outcome six months after treatment initiation.

In order to evaluate how good our backward regression model fits our data, the Akaike information criterion (AIC) was estimated at a low value of 69.02, suggesting a low estimated prediction error of the statistical model.

**Figure 7:**
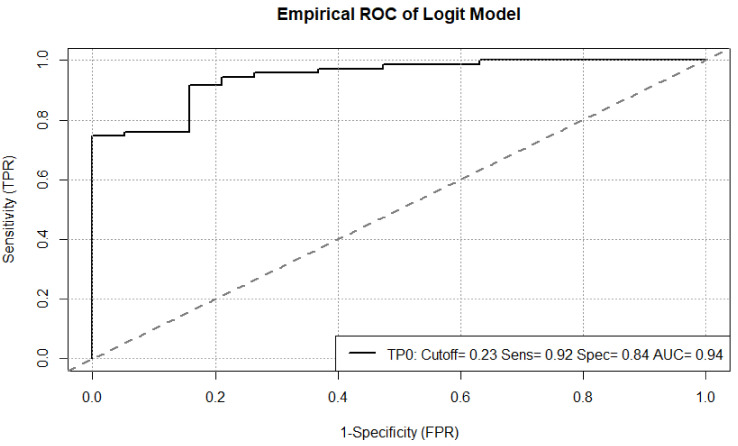
Receiver operating characteristic ROC of our backward regression model for outcome prediction in metastatic melanoma patients six months after initiation of immunotherapy.

Furthermore, we assessed the performance of our backward regression model for outcome prediction six months after initiation of immunotherapy based on our definition, i.e., clinical benefit vs. no clinical benefit as endpoint. For this purpose, a receiver operating characteristic ROC was used displaying a sensitivity (TPR) of 0.92, specificity (FPR) of 0.84 and an area under the curve AUC of 0.94 (Figure 7).

## 4. Discussion

The value of semiquantitative parameters on FDG-PET/CT scans to predict treatment response has been extensively discussed in the literature for several malignancies albeit with a lack of evidence in large cohorts of melanoma patients [11,12,13,14,15].

We aimed to investigate whether metastatic volume and semiquantitative parameters on FDG-PET/CT scans performed at baseline TP 0 and three months after initiation of immunotherapy TP 1 can be used at metastasis- and patient-level to predict the outcome six months after initiation of immune checkpoint inhibition TP 2 in a large cohort of metastatic melanoma patients.

First of all, our results provided evidence at metastasis-level, that a vast majority of melanoma metastases did respond to immune checkpoint inhibition in the first six months since only 11.8 % of all metastases at TP 2 were progressive vs. 88.2% non-progressive metastases with clinical benefit.

Our results also suggested that the initial volume of melanoma metastases shouldn’t be used for treatment outcome prediction at metastasis-level, given similar median values of volume between progressive metastases and non-progressive metastases with clinical benefit at baseline. However, initial SUVmean and SUVpeak of melanoma metastases can be indicative of treatment response or resistance at metastasis-level but shouldn’t be considered alone given the lower prediction power.

Our results provided strong evidence that metastatic SUVpeak and early volume changes have a strong power to predict the response to therapy at metastasis-level.

Clinical parameters can also be taken into account as part of the analysis at metastasis-level. Although bone metastasis only represented 7% of all included metastasis at TP 0, their presence seemed to have a negative influence on the outcome at TP 2, especially in women showing the lowest likelihood of clinical benefit compared to other metastasis entities. Also, the occurrence of new metastases at TP 1 seemed to be a negative predictive biomarker.

The prognostic power of semiquantitative parameters in context of melanoma has been controversial discussed in recent investigations mostly performed in smaller cohorts.

In a retrospective review published in 2016 including 41 melanoma patients pretreatment SUVmax was found to be significantly higher in non-survivors than in survivors, but also higher in patients with recurrence than patients without recurrence. Besides the smaller cohort, Son et al. measured SUVmax, metabolic tumor volume MTV and total lesion glycolysis TLG of the primary tumor, while we exclusively considered metastatic parameters since the primary tumor was already resected when the pretreatment FDG-PET/CT scan was performed to detect distant metastasis. Furthermore, the authors assessed treatment outcome using disease-free-survival DFS and melanoma-specific-survival MSS following all included patients over five years [14], while we defined clinical benefit and no clinical benefit based on FDG-PET/CT scans performed six months after treatment.

A retrospective study published in 2019 including 55 melanoma patients reported that SUVmax was not predictive of outcome. Semiquantitative parameters (SUVmax, SUVmean, MTV and TLG) were not manually as we did but automatically measured using a software for each included lesion. Furthermore, the treatment outcome after single immune checkpoint inhibition with anti-PD1 only was defined by the overall survival OS, progression free survival PFS and best overall response BOR with a median follow-up of 20 months [13], while we focused on early treatment outcome six months after start of single- or double-checkpoint inhibition based on FDG-PET/CT scans only.

In 2020 Dittrich et al. investigated retrospectively in 26 melanoma patients treated either with vemurafenib (*n* = 9) or ipilimumab (*n* = 17) the prognostic power of standardized uptake values [15]. In contrast to our methodology standardized uptake values were measured automatically and not manually for each included lesion on FDG-PET/CT scans pretreatment and 12 weeks after treatment start. SUVmax and SUVmean showed in patients treated with vemurafenib only a significant prediction power [15].

However, an additional analysis on a patient-level appeared clinically more relevant, as part of the discussion in the interdisciplinary tumor board.

Our results provided evidence that 90 % of all included metastatic melanoma patients (*n* = 101) benefited from immune checkpoint inhibition six months after treatment start (i.e., no disease progression under treatment), while 10 % displayed progressive disease at the same time. Furthermore, total tumor volume and number of metastases at baseline did not show any predictive power, suggesting that metastatic melanoma patients with higher tumor load initially can still benefit from immunotherapy.

Three months after treatment initiation, total tumor volume, total MTV and TLG of all metastases can be used as strong negative predictive biomarkers for treatment response six months after treatment start. Interestingly, the occurrence of immune-related adverse events during the first six months of immunotherapy did not show any predictive power for early response, implying, that patients presenting early immune related side effects can still benefit from the ongoing treatment. Also, our results provided strong evidence, that early complete response on a metastasis- and patient-level seems to be predictive for lasting complete response. Tan et al. observed in their investigations published in 2018, that a small proportion of all included patients (*n* = 104) reached a complete response at one year [16]. However, the vast majority of these patients had long lasting response to therapy (median follow-up 30.1 months) [16]. Interestingly, early progressive disease on a metastasis- and patient-level three months after treatment start does not exclude subsequent clinical benefit under ongoing immune checkpoint inhibition, since some patients initially displaying progressive disease three months after treatment initiation showed a stable disease to complete response three months later. Furthermore, bone metastases were negative predictors for response. These results are in accordance with recent investigations published in 2020. Da Silva et al. reported that different anatomical metastatic locations may display different response patterns and so influence overall response and survival under dual immunotherapy, highlighting the importance of personalized treatment [17].

Finally, clinical parameters, such as age and female sex were also negative predictive biomarkers with lower prediction power.

Our results bring innovative and clinically relevant insights to light in knowledge of the current controversial literature. Besides the important size of our cohort, our results provide strong evidence, a prediction of early response to immune checkpoint inhibition using FDG-PET/CT is possible with a low estimated prediction error. Additionally, we have highlighted the importance of an accurate timing for outcome prediction since we have reported a different prediction power pretreatment TP 0 and during treatment TP 1 at metastasis- and patient-level.

The main limitation of this study includes its single-center retrospective design with images acquired on different PET/CT scanners from 2013 to 2019. Four different PET/CT scanners by GE were used over time, i.e., Discovery ST16 und VCT (2013–2017), Discovery 690 (2013–2019) and Discovery DMI (2017–2019), which could have influenced the measurement of PET semiquantitative parameters, despite calibration and cross-calibration of the used scanners.

These results will be confronted with long-term outcome results (based on overall survival OS and progression free survival PFS) in a larger population of metastatic melanoma patients in a separate investigation in kind cooperation with our department of dermatology.

## 5. Conclusions

FDG-PET/CT scans performed for treatment response assessment three months after initiation of immune checkpoint inhibition in metastatic melanoma patients can be used to predict early response to treatment. On a metastasis-level SUV peak and volume variation of metastases are strong outcome predictive biomarkers. On a patient-level total tumor volume and semiquantitative parameters such as total metabolic tumor volume MTV and total lesion glycolysis TLG of all metastases are promising outcome predictive biomarkers. Also, early complete response on a metastasis- and patient-level seems to be predictive for lasting complete response.

## Figures and Tables

**Table 1:** Baseline characteristics of all included metastatic melanoma patients including gender, median age, histopathology of primary tumor, prior treatment at TP 0, type of checkpoint inhibition from TP 0 to TP 2 and anatomical site of melanoma metastasis at TP 0.

Characteristics	*n* (In Percentage %)
**Patient cohort**	
Total	111(100%)
Male	77 (69.4%)
Female	34 (30.6%)
Median age in years (interquantile range)	69 (55-76)
**Histopathology of primary tumor**	
Superficial spreading	33 (29.7%)
Nodular	28 (25.3%)
Lentigo maligna	9 (8.1%)
Acral lentiginous	9 (8.1%)
Sinonasal	1 (0.9%)
Mucosal	5 (4.5%)
Amelanotic	6 (5.4%)
Eye melanoma	3 (2.7%)
Unknown primary	17 (15.3%)
**Prior treatment at TP 0**	
Native	43 (38.7%)
Pretreated	68 (61.3%)
Ipilimumab	52 (76.5%)
Ipilimumab + Nivolumab	3 (4.4%)
BRAF-Inhibitor	2 (2.9%)
MEK-Inhibitor	4 (5.9%)
Chemotherapy	7 (10.3%)
**Checkpoint inhibition from TP 0 to TP 2**	
Single	94 (84.7%)
Dual	17 (15.3%)
**Anatomical site of metastasis at TP 0**	
1 = soft tissue	319 (52.1%)
2 = lung	137 (22.4%)
3 = liver/spleen	111 (18.1%)
4 = bone	45 (7.4%)

## Data Availability

No new data were created or analyzed in this study. Data sharing is not applicable to this article.

## Abbreviations

3D	Three-dimensional
ANOVA	Analysis Of Variance
Anti-CTLA4	Anti-Cytotoxic T-Lymphocyte-associated Protein 4
Anti-PD1	Anti-Programmed Cell Death 1
BMI	Body-Mass-Index
BOR	Best Overall Response
CB	Clinical Benefit
CR	Complete Response
CT	Computed Tomography
DFS	Disease Free Survival
FDG-PET/CT	^18^F*-*2-Fluoro-2-desoxy-D-glucose Positron Emission Tomography/Computed Tomography
GCP	Good Clinical Practice
GE	General Electric
h	Hour
ICI	Immune Checkpoint Inhibitors
IQR	Interquartile Range
kV	Kilovolt
LDH	Lactate Dehydrogenase
mA	Milliampere
mg/dL	Milligram/Deciliter
mL	Milliliter
MSS	Melanoma Specific Survival
MTV	Metabolic Tumor Volume
OSEM	Ordered Subset Expectation Maximization
PD	Progressive Disease
PET	Positron Emission Tomography
PFS	Progression Free Survival
PR	Partial Response
S100	Calcium-binding protein B
SD	Stable Disease
SUV	Standardized Uptake Value
TLG	Total Lesion Glycolysis
TOF	Time-of-Flight
TP	Time Point
VOI	Volume of Interest
