# Correction: Kudura et al. Prediction of Early Response to Immune Checkpoint Inhibition Using FDG-PET/CT in Melanoma Patients. *Cancers* 2021, *13*, 3830

**DOI:** 10.3390/cancers14133268

**Published:** 2022-07-04

**Authors:** Ken Kudura, Florentia Dimitriou, Lucas Basler, Robert Förster, Daniela Mihic-Probst, Tim Kutzker, Reinhard Dummer, Joanna Mangana, Irene A. Burger, Michael C. Kreissl

**Affiliations:** 1 Department of Nuclear Medicine, University Hospital Zurich, 8091 Zurich, Switzerland; irene.burger@usz.ch; 2 Department of Dermatology, University Hospital Zurich, 8091 Zurich, Switzerland; florentia.dimitriou@usz.ch (F.D.); reinhard.dummer@usz.ch (R.D.); johanna.mangana@usz.ch (J.M.); 3 Center for Proton Therapy, Paul Scherrer Institute, 5232 Villigen, Switzerland; lucas.basler@psi.ch; 4 Institute of Radiation Oncology, Cantonal Hospital Winterthur, 8400 Winterthur, Switzerland; robert.foerster@ksw.ch; 5 Institute of Pathology and Molecular Pathology, University Hospital Zurich, 8091 Zurich, Switzerland; daniela.mihic@usz.ch; 6 Faculty of Applied Statistics, Humboldt University Berlin, 10117 Berlin, Germany; tim.kutzker@hu-berlin.de; 7 Department of Nuclear Medicine, Cantonal Hospital Baden, 5404 Baden, Switzerland; 8 Department of Radiology and Nuclear Medicine, Division of Nuclear Medicine, University Hospital Magdeburg, 39120 Magdeburg, Germany; michael.kreissl@med.ovgu.de

The authors wish to make the following corrections to this paper [1]:

1. Add a citation in the Introduction section

In the original publication, the publication from Lucas Basler et al. [2] was not cited. The citation has now been inserted in the Introduction as reference [9]:

In order to predict the response to immunotherapy several outcome predictive biomarkers based on the histopathology of the primary tumor (i.e., tumor thickness, ulceration, mitotic rate) [8], standard blood samples (i.e., lactate dehydrogenase LDH, calcium-binding protein B S100B) [9] or clinical aspects (i.e., sentinel lymph node involvement, anatomical site of primary tumor, gender or age) [10] have been already discussed in the literature.”

2. Ethical approval information needs to be updated:

The official and correct reference of the stated ethical approval is KEK-ZH-Nr: 2014-0193. 

The correct statement is as below:

**Institutional Review Board Statement**: The study was conducted in accordance with the Declaration of Helsinki and approved by the Cantonal Ethics Committee of the Canton Zurich in Switzerland (protocol code KEK-ZH-Nr: 2014-0193) on 15 March 2017. 

*2.1. Patient Cohort*

(…). All included patients consented the use of their clinical data for research purposes. This study was approved by the local ethics committee (protocol code KEK-ZH-Nr: 2014-0193) and conducted in compliance with Good Clinical Practice GCP-rules and the Declaration of Helsinki.

3. Missing Funding

In the original publication, the funder **Cancer Research Center, Comprehensive Center Zurich, University Hospital Zurich (CRC_13)** was not included. 

The correct statement is as:

**Funding**: This research was funded by the Iten-Kohaut-Foundation (IA USZF27071) and the Cancer Research Center, Comprehensive Center Zurich, University Hospital Zurich (CRC_13).

The authors apologize for any inconvenience caused and state that the scientific conclusions are unaffected. The original publication has also been updated.
